# PP2A-Mediated Dephosphorylation of p107 Plays a Critical Role in Chondrocyte Cell Cycle Arrest by FGF

**DOI:** 10.1371/journal.pone.0003447

**Published:** 2008-10-17

**Authors:** Victoria Kolupaeva, Emmanuel Laplantine, Claudio Basilico

**Affiliations:** Department of Microbiology, New York University School of Medicine, New York, New York, United States of America; University of Hong Kong, China

## Abstract

FGF signaling inhibits chondrocyte proliferation, a cell type-specific response that is the basis for several genetic skeletal disorders caused by activating FGFR mutations. This phenomenon requires the function of the p107 and p130 members of the Rb protein family, and p107 dephosphorylation is one of the earliest distinguishing events in FGF-induced growth arrest. To determine whether p107 dephoshorylation played a critical role in the chondrocyte response to FGF, we sought to counteract this process by overexpressing in RCS chondrocytes the cyclin D1/cdk4 kinase complex. CyclinD/cdk4-expressing RCS cells became resistant to FGF-induced p107 dephosphorylation and growth arrest, and maintained significantly high levels of cyclin E/cdk2 activity and of phosphorylated p130 at later times of FGF treatment. We explored the involvement of a phosphatase in p107 dephosphorylation. Expression of the SV40 small T-Ag, which inhibits the activity of the PP2A phosphatase, or knockdown of the expression of the PP2A catalytic subunit by RNA interference prevented p107 dephosphorylation and FGF-induced growth arrest of RCS cells. Furthermore, an association between p107 and PP2A was induced by FGF treatment. Our data show that p107 dephosphorylation is a key event in FGF-induced cell cycle arrest and indicate that in chondrocytes FGF activates the PP2A phosphatase to promote p107 dephosphorylation.

## Introduction

The formation and growth of long bones and vertebrae is achieved through endochondral ossification, a strictly regulated process that requires the proliferation and differentiation of chondrocytes. Chondrocytes commitment, proliferation, and differentiation are regulated by a number of signaling molecules and transcription factors, and genetic and biochemical studies have identified FGF signaling as a central regulator of these processes [1]. The excessive or unregulated FGF signaling caused by activating FGFR mutations strongly inhibits chondrocyte proliferation and affects their differentiation, resulting in several bone morphogenetic disorders, the best known of which is achondroplasia [2].

The inhibition of chondrocyte proliferation induced by FGF is a cell type-specific response that contrasts with the mitogenic effect of FGFs in most other cells, raising the question of how the same signaling inputs are interpreted by different cell types to generate specific patterns of gene expression and biological response. In chondrocytes, FGFs activate a complex network of signaling and transcriptional events that result in growth inhibition and the induction of some aspects of differentiation [3]. In studies aimed at identifying the key effector molecules and events responsible for the FGF-induced growth-arrest, we had previously established that it requires the function of the p107 and p130 members of Retinoblastoma (Rb) family, but not of pRb [4], in line with the observation that p107/p130 knockout mice exhibit exaggerated chondrocyte proliferation [5]. Furthermore, cell and organ culture experiments suggested that p107 played the major role in the FGF response[4]. Rb proteins are essential cell cycle regulators and their function is regulated by phosphorylation at several Ser/Thr residues. In the active hypophosphorylated form, Rb proteins interact with and inhibit transcriptional activation by the E2F family of transcription factors that control the expression of many cycle progression genes. Phosphorylation by cyclin dependent kinases (cdk) inactivates the Rb proteins, allowing E2F factors to positively influence the transcription of cell cycle genes and cell proliferation [6]. Consistent with the growth inhibitory response, p107, p130 and pRb all become dephosphorylated upon FGF treatment of chondrocytes, but while p130 and pRb undergo dephosphorylation 10–12 hours after exposure of the cells to FGF, p107 is dephosphorylated very rapidly (0.5–1 hour). p107 dephosphorylation is also observed in the presence of RNA and protein synthesis inhibitors, indicating that it results from a signaling event, while dephosphorylation of p130 and pRb requires the induction of gene transcription [3]. Furthermore, the finding that p107 dephosphorylation occurred while chondrocytes still exhibited robust activity of cyclin/cdk complexes and prior to the induction of cdk inhibitors [3], [7] suggested that it resulted from the activation of a phosphatase.

While the results mentioned above suggested that p107 dephosphorylation played an important role in FGF-induced cell cycle arrest, it is known that ectopic activation of Rb downstream effectors, such as E2F-1, c-myc or cyclin E can bypass Rb regulation and promote cell proliferation in the presence of hypophosphorylated Rb proteins [8]–[10]. Thus FGF signaling could conceivably affect other molecules regulating cell cycle progression and p107 dephosphorylation could have been irrelevant to growth arrest and/or secondary to some feedback mechanisms initiated by FGF. We have therefore investigated whether p107 dephosphorylation was a critical event in the growth-inhibitory response of chondrocytes to FGF. The results presented here show that preventing p107 dephosphorylation by the overexpression of cyclin D1/cdk4 complexes in RCS chondrocytes abolishes the growth suppression exerted by FGF. To determine whether a specific cellular phosphatase was responsible for p107 dephosphorylation, we expressed the SV40 small T-Ag (ST), which is known to inactivate the PP2A phosphatase, in RCS cells. ST expression prevents FGF-induced p107 dephosphorylation and growth arrest, and similar results are obtained by knock-down of PP2A expression through shRNA. Furthermore, coimmunoprecipitation experiments show that an association between p107 and PP2A is induced in RCS cells shortly after FGF treatment. We conclude that p107 dephosphorylation is an early and critical event in the FGF response of chondrocytes that is mediated by PP2A. These results place PP2A upstream of p107 dephosphorylation, adding a novel pathway to the FGF signaling network.

## Results

### Overexpression of the cyclin D1/cdk4 complex prevents p107 dephosphorylation and cell cycle arrest in FGF-treated RCS chondrocytes

In the normal cell cycle, Rb proteins are phosphorylated by the G1 cyclin D/cdk4 (6) and cyclin E/cdk2 kinase complexes [11], [12]. While cyclin E/cdk2 can target both p107 and p130, biochemical and genetic evidence indicates a specific requirement for cyclin D1/cdk4 complexes for p107 inactivation. Indeed, coexpression of cyclin D1 and cdk4, but not of the cyclin E/cdk2 complex, can reverse a p107-induced cell cycle block [13]–[20]). To determine whether p107 dephosphorylation played an important role in the chondrocyte response to FGF, we therefore thought to counteract the FGF-induced dephosphorylation of p107 by overexpressing the cyclin D1/cdk4 complex. We generated RCS chondrocytic cell lines constitutively expressing relatively high levels of cyclin D1 and cdk4 and tested how this affected p107 dephosphorylation and the growth inhibitory FGF response. Although p107 is also rapidly dephosphorylated upon FGF treatment of murine growth plate primary chondrocytes (Fig. S1), RCS cells were chosen for these experiments, as they exhibit essentially all properties of proliferating chondrocytes and allow genetic experiments that cannot be performed in primary chondrocytes, whose life span is very limited. Of note, we had previously shown (4) that p107 and p130 were essential FGF effectors also in RCS cells.

**Figure 1:**
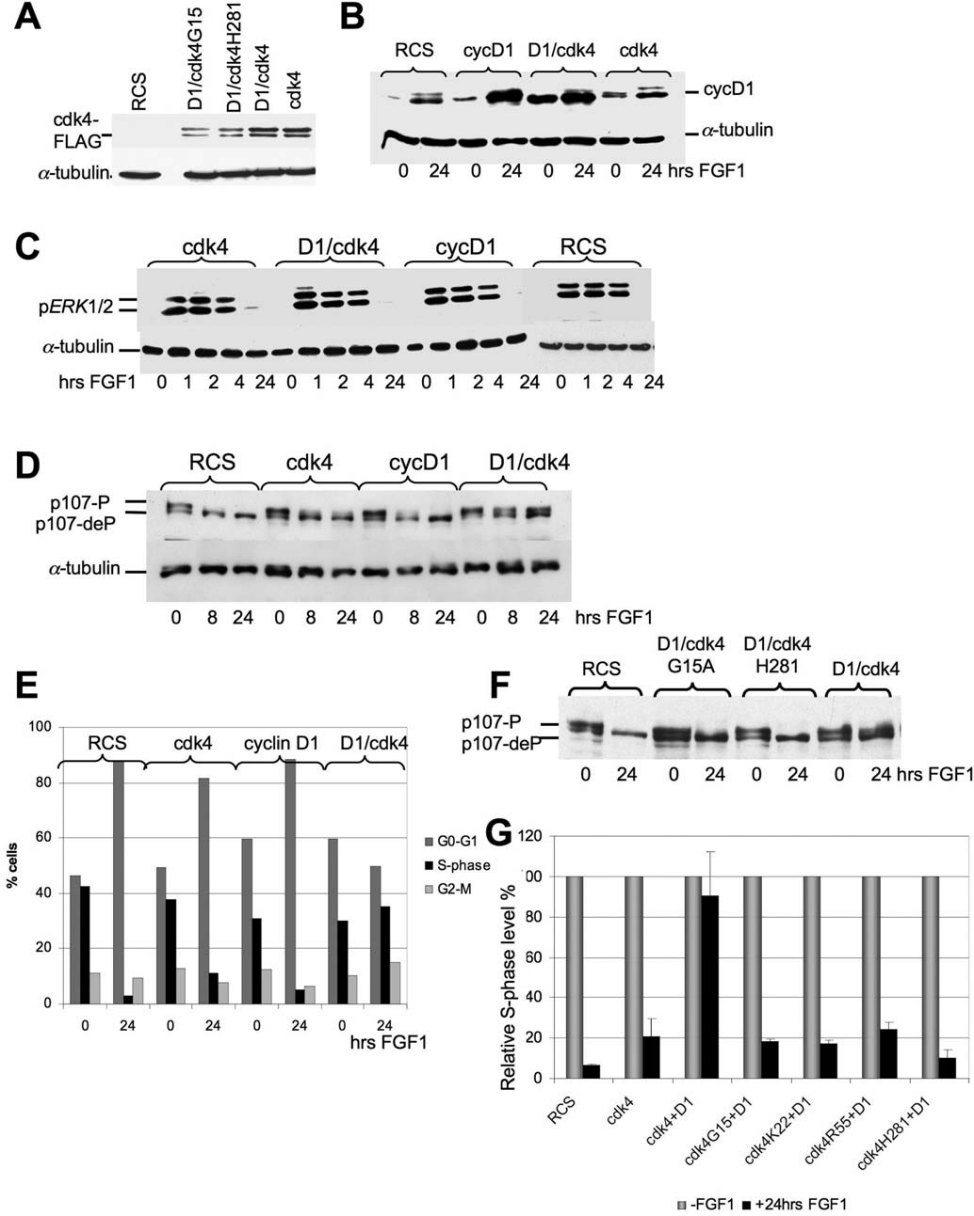
Overexpression of an active cyclin D1/cdk4 complex prevents p107 dephosphorylation and cell-cycle arrest in FGF-treated RCS. Cyclin D1 and cdk4 were stably introduced into RCS cells either independently or in combination and these cell lines were treated with FGF1 for the indicated periods of time. 20 µg of total protein from all indicated cell lines were analyzed by SDS-PAGE followed by immunoblotting. Equal amount of protein loading was confirmed by α-tubulin immunodetection. (A) Anti-FLAG antibodies identify two bands in the cell lines overexpressing FLAG-tag cdk4. Both bands are equally recognized by anti-cdk4 antibody (data not shown). (B) Cyclin D1 is upregulated upon FGF treatment in all cell lines. (C) ERK1/2 phosphorylation is activated in a similar manner in all experimental cell lines. (D, F) Western blots with anti-p107 antibodies. Hyper-and hyposphosphorylated forms of p107 are indicated. (E, G) FACScan™ analysis of RCS cells overexpressing cdk4, cyclin D1 or cyclin D1 and wild type or mutant cdk4 in response to FGF treatment. (E) Numbers on the Y-axis indicate percentage of total cells in G1, S and G2-M phases. (G) In all mutant forms of cdk4 the indicated amino acids were substituted by alanine. Changes in the levels of S-phase cells are indicated. Several clones of each cell type were used with essentially identical results.

Retroviral vectors expressing cdk4 (FLAG-tagged) or cyclin D1 were introduced into RCS cells individually or in combination. Cells were selected with an appropriate antibiotic and the level of expression was determined by anti-cyclin D1, anti-cdk4, and anti-FLAG antibodies (Fig. 1 A, B). Clones expressing moderately high levels of cyclin D1 and cdk4 were selected for further analysis. Three clones overexpressing both cyclin D1 and cdk4 and two clones from either cyclin D1 or cdk4 overexpressing cells were tested for their response to FGF.

Since activation of ERK1/2 MAP kinases contributes to the FGF-induced growth arrest of chondrocytes and appears to be required for p107 dephosphorylation [21], we first determined whether these signal transduction pathways were normally activated in our cell lines. Fig. 1C shows that a strong and sustained activation of ERK1/2 was induced by FGF in the parental RCS cells as well as in cells overexpressing cdk4, cyclin D1, or both cyclin D1 and cdk4, indicating that FGF signal transduction is intact in these cell lines.

We then determined whether cyclin D1 and cdk4 overexpression affected p107 phosphorylation and proliferation in FGF-treated cells. Exponentially growing RCS cells contain a mixture of hyper-and hypo-phosphorylated p107, which is completely converted to the hypophosphorylated form within 1 hr of FGF treatment, and remains dephosphorylated up to 24 hrs [3]. Similar results were observed in the cell lines overexpressing cyclin D1 and cdk4 independently. In contrast, p107 was still hyperphosphorylated after 24 hrs of FGF treatment when cyclin D1 and cdk4 were coexpressed (Fig. 1D). Furthermore, overexpression of both cyclin D1 and cdk4 clearly blocked FGF-mediated growth arrest of RCS cells, as assayed by FACScan analysis (Fig. 1E), while introduction of either cyclin D1 or cdk4 alone had no significant effect. We also determined how cell proliferation was affected by FGF treatment in RCS cells overexpressing cyclin D1 and cdk4 cells. As expected, while the parental RCS cells ceased to proliferate within 10–12 hours, cdk4/D1 expressing cells continued to proliferate and increase in number (data not shown).

To support the conclusion that an enzymaticaly active cyclin D1/cdk4 complex is necessary to overcome growth inhibition by FGF we overexpressed several mutant forms of cdk4 defective in cyclin D1 binding together with wild type cyclin D1 (Fig. 1A, F). All mutants were characterized by great reduction in kinase activity toward Rb-GST fusion protein as assayed *in vitro* [22], [23]. We introduced a Lys22 to Ala (cdk4K22A) mutation and a triple mutation His281, Lys282, Arg283 to Ala (cdk4H281-3A). Both mutations also affected binding of p16INK4a cdk inhibitor (p16) to cdk4, since cyclin D1 and p16 have overlapping binding sites on cdk4. We also introduced two mutants (cdk4G15A and cdk4R55A) that are defective in binding to cyclin D1, but still retain high affinity for the p16. Expression of all of these mutants did not prevent the FGF-induced dephosphorylation of p107 (Fig. 1F and data not shown) and FACScan analysis showed that these cell lines were growth-inhibited upon FGF treatment (Fig. 1G), indicating that an active D1/cdk4 complex is required to overcome the FGF-inhibitory effect. We can also exclude that overexpressed D1/cdk4 complexes simply sequester p16 because the cdk4G15A and cdk4R55A mutants were still unable to prevent FGF-mediated growth arrest.

In conclusion, these results indicate that constitutive overexpression of the cyclinD1/cdk4 complex in RCS cells blocks FGF-induced p107 dephosphorylation and growth arrest, consistent with the hypotheses that p107 dephosphorylation is a crucial initial event in the response of chondrocytes to FGF signaling. Interestingly cyclin D1 expression is significantly induced by FGF in RCS cells [3], (Fig. 1B), but overexpression of cdk4 alone has only a minimal effect on p107 dephosphorylation and growth arrest. Since cyclin D1 is induced with relatively slow kinetics, it is likely that a high D1/ cdk4 activity has to be present at the time of first exposure to FGF, to prevent the cascade of events initiated by p107 dephosphorylation.

### Overexpression of cyclin D1 and cdk4 also affects later events in the FGF response

**Figure 2:**
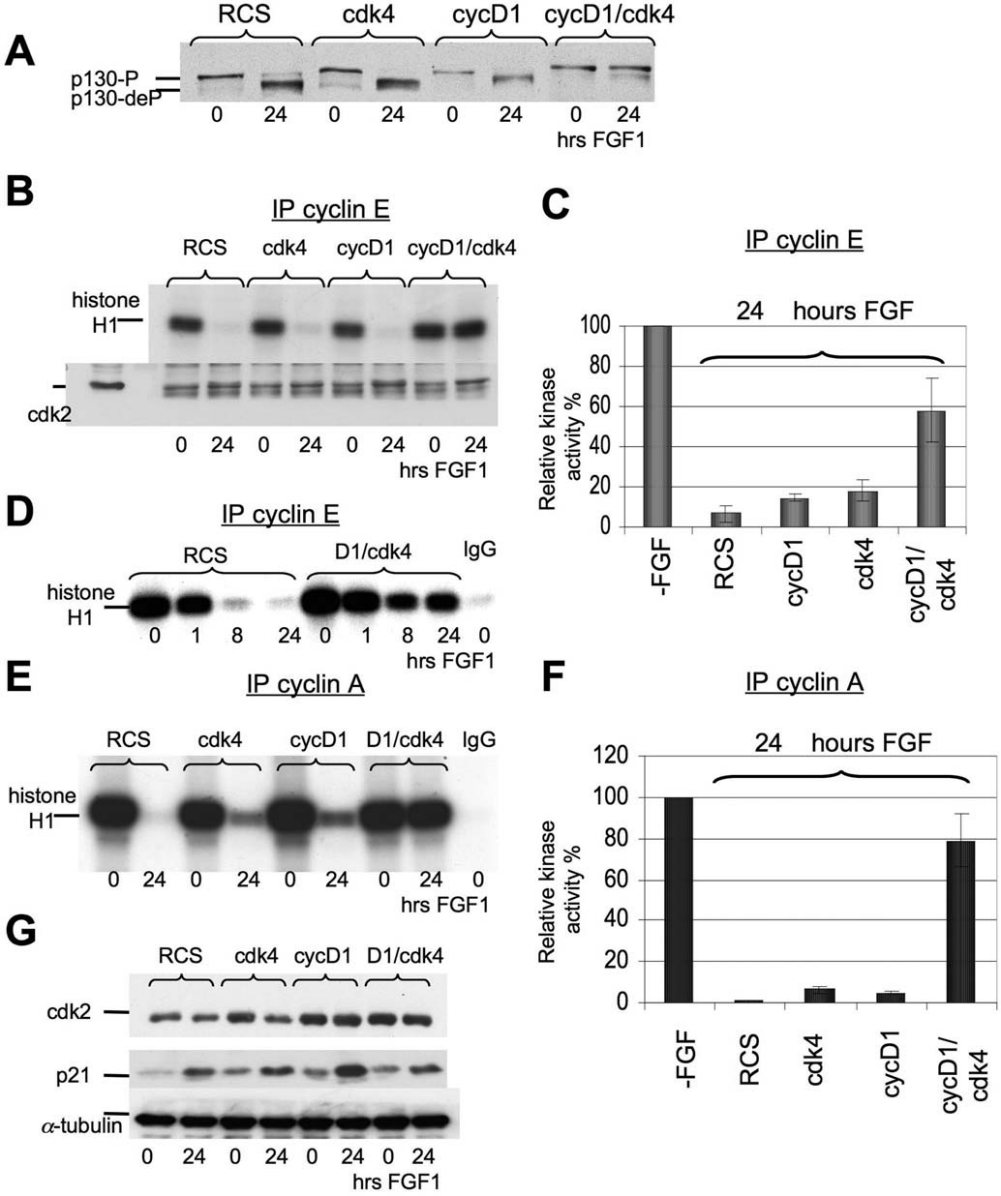
Effect of cyclin D1/cdk4 overexpression on p130 phosphorylation and cyclin E/cdk2 and cyclin A/cdk 2 kinase activities. Parental RCS, cyclin D1, cdk4 or cyclin D1/cdk4 cell lines were treated with FGF1 for the indicated times. (A) p130 dephosphorylation is prevented in cyclin D1/cdk4 expressed cells. 100 µg of total protein was analyzed by SDA-PAGE followed by immunoblotting with anti p130-antibodies. (B, D, E) Kinase activity of immunoprecipitated cyclin E/cdk2 and cyclin A/cdk 2 complexes as assayed *in vitro*. The cyclin E/cdk2 and cyclin A/cdk 2 complexes were isolated using anti-cyclin E and anti-cyclin A antibodies and histone H1 was used as a substrate. Antibody to mouse IgG was used as negative control. Equal amount of protein loading was confirmed by immunodetection of cdk 2 in immunoprecipitated complexes. (C, F) Summary of results from 3 independent experiments depicting relative levels of cyclinE/cdk2 and cyclin A/cdk2 activities upon FGF treatment in different cell lines. (G) 20 µg of total protein were analyzed by WB, with antibodies against cdk2 or p21. Equal amount of protein loading was confirmed by α-tubulin immunodetection.

The maintenance of FGF-induced growth arrest is characterized by the accumulation of hypophosphorylated p130 by 10–16 hrs of FGF treatment, that follows a dramatic drop in cyclin E/cdk2 activity between 6 and 12 hrs [3]. We therefore investigated the phosphorylation status of p130 in the cells overexpressing both cdk4 and cyclin D1 after FGF treatment. p130 also remained phosphorylated in this cell line, while FGF caused dephosphorylation of p130 when cdk4 or cyclin D1 were overexpressed independently (Fig. 2A.) Several reports [16], [18], [19] indicate that the cyclin D1/cdk4 complex can phosphorylate both p107 and p130, but that inactivation of p130 requires the phosphorylation of additional sites by the cyclin E/cdk2 complex. We therefore studied the activity of the cyclin E/cdk2 complex in our cell lines.

Anti-cyclin E antibody was used to immunoprecipitate cyclin E/cdk2 complexes from protein lysates derived from untreated cells or cells treated with FGF for 24 hrs. The immunoprecipitated complexes were then tested for their ability to phosphorylate histone H1 substrate *in vitro*. As shown in Fig 2B–D, after 24 hrs of the FGF treatment cyclin E/cdk2 activity was drastically reduced in FGF-treated RCS cells, cyclin D and cdk4 cells, but D1/cdk4 cells retained about 60% of cyclinE/cdk2 activity. A time course experiment (Fig. 2D) showed that after 1 hr of FGF treatment RCS cells retained most cyclinE/cdk2 kinase activity, similar to D1/cdk4 cells. However, after 8 hrs of FGF treatment the cyclin E/cdk2 kinase activity of RCS cells dropped dramatically, becoming essentially nil at 24 hrs, while D1/cdk4 cells retained substantial cyclinE/cdk2 activity, even after 24 hrs of FGF treatment. Since cdk2 also complexes with cyclin A[11], which is induced in S phase slightly later than cyclin E, we tested the activity of cyclin A/cdk2 complexes. Similarly to cyclin E/cdk2, cyclin A/cdk activity was strongly reduced by FGF treatment in the parental RCS cells, but remained high in the D1/cdk4 cells (Fig. 2 E, F).

FGF treatment of RCS cells is accompanied by down regulation of cdk2 and up regulation of the cdk2 inhibitor p21[3], [7]. Fig. 2G shows that the level of cdk2 expression in D1/cdk4 cells after 24 hrs of FGF treatment was slightly elevated compared to RCS, cdk4 or cyclin D1 RCS cell lines, while the increase in p21 expression was undistinguishable among experimental cell lines (Fig. 2G). Thus these results indicate that overexpression of active cyclinD1/cdk4 complexes not only prevents the dephosphorylation of p107 but also allows higher sustained levels of cyclinE/cdk2 activity, that may contribute to maintaining p107 and p130 in the hyperphosphorylated state as well as cell cycle progression.

### PP2A inhibition prevents p107 dephosphorylation and FGF-induced cell cycle arrest

The regulation of Rb protein family phosphorylation by cyclin/cdk kinases is fairly well understood, but much less is known about the phosphatases that dephosphorylate Rbs during the normal cell cycle or in response to exogenous signals. Since previous results [3], [7] indicated that p107 dephosphorylation occurred when FGF-treated chondrocytes still express robust cyclin/cdk activity, we investigated the hypothesis that the induction of p107 dephosphorylation by FGF resulted from the activation of a phosphatase[3]. We immunoprecipitated p107 from FGF-treated and untreated RCS cells and measured p107-associated serine/threonine phosphatase activity. Immunoprecipitates of FGF-treated cells showed a two-three-fold increase in phosphatase activity compared to controls (discussed later), consistent with the hypothesis that FGF targets a phosphatase to p107.

**Figure 3:**
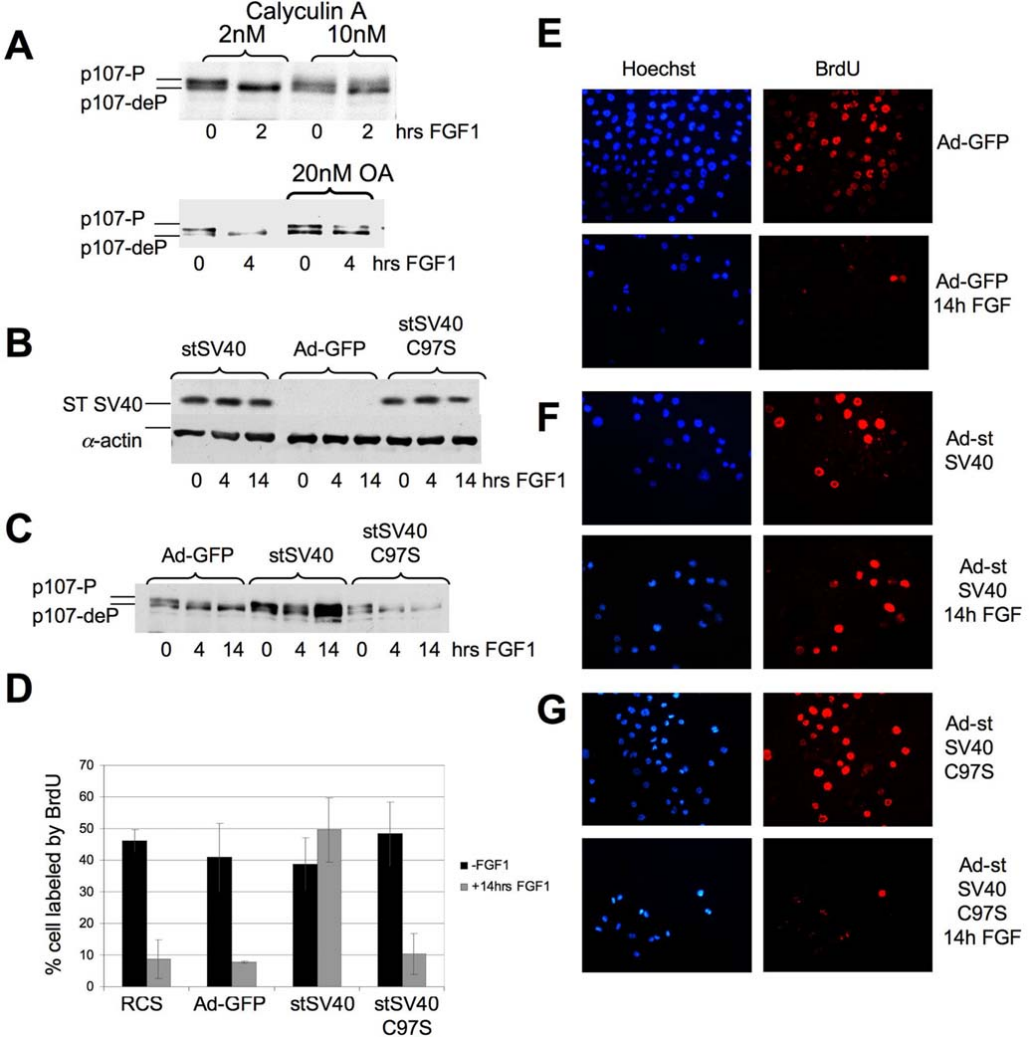
FGF-induced dephosphorylation of p107 is prevented by protein phosphatase inhibitors and by SV40 small t-antigen (st SV40). (A) RCS cells were pretreated with either okadaic acid (OA) or Calyculin A 2 hours before FGF treatment. 20 µg of total protein were subjected to SDS-PAGE following inmmunodetection of p107. (B–G) RCS cells were infected with adenoviruses expressing GFP, st SV40 or st SV40 mutant C97S following FGF1 treatment as indicated. (B,C) 20 µg of total protein were used for immmunodetection of (B) st SV40 and (C) p107. Equal amount of protein loading was confirmed by α-actin immunodetection. (D–G) BrdU incorporation assay of FGF treated or untreated RCS cells expressing either GFP, st SV40, or st SV40 C97S mutant. BrdU was detected by using BrdU antibodies (red). Nuclei were stained with DAPI (blue). (D) Summary of results in panels E–G.

We then determined whether inhibition of phosphatase activity affected the p107 status in FGF-treated RCS cells, using okadaic acid (OA) or calyculin A which are potent inhibitors of protein phosphatases [24]. As shown in Fig. 3A, p107 dephosphorylation was significantly prevented in the presence of OA, or calyculin A.

PP2A is an abundant and ubiquitous Ser/Thr phosphatase that plays a role in numerous signaling pathways [25] and has been implicated in regulation of Rb-protein phosphorylation [26]–[30]. PP2A has a trimeric structure consisting of a catalytic C subunit, a scaffolding A subunit (PR65), and a variable third regulatory B subunit which is represented by 4 different families, including at least twenty four different proteins, that play a role in substrate recognition and subcellular localization [31]. PP2A activity can be blocked *in vivo* and *in vitro* by chemical inhibitors, such as OA and calyculin A, or through interaction of PP2A with the small T-antigen (ST) of SV40 virus [32], [33], that forms a stable complex with the A subunit, thereby competing with regulatory B subunits.

To determine if PP2A plays a role in the FGF-induced dephosphorylation of p107, we infected RCS cells with adenovirus vectors overexpressing GFP, ST or a mutant form of ST (Cys97Ser) [34] which is unable to bind and inhibit PP2A (Fig. 3B). Expression of ST inhibited p107 dephosphorylation in FGF treated cells almost completely, while the mutant form of ST failed to prevent it (Fig. 3C). As shown by BrdU incorporation, FGF caused strong inhibition of DNA synthesis in RCS cells and in Ad-GFP cells (Fig. 3D, E). However, adenovirus-driven expression of ST overcame the FGF growth inhibitory effect (Fig. 3D, F) which was not the case when the mutant form of ST was expressed (Fig. 3D, G). These results strongly suggest that PP2A plays an essential role in the dephosphorylation of p107 and the cell cycle arrest induced by FGF.

**Figure 4:**
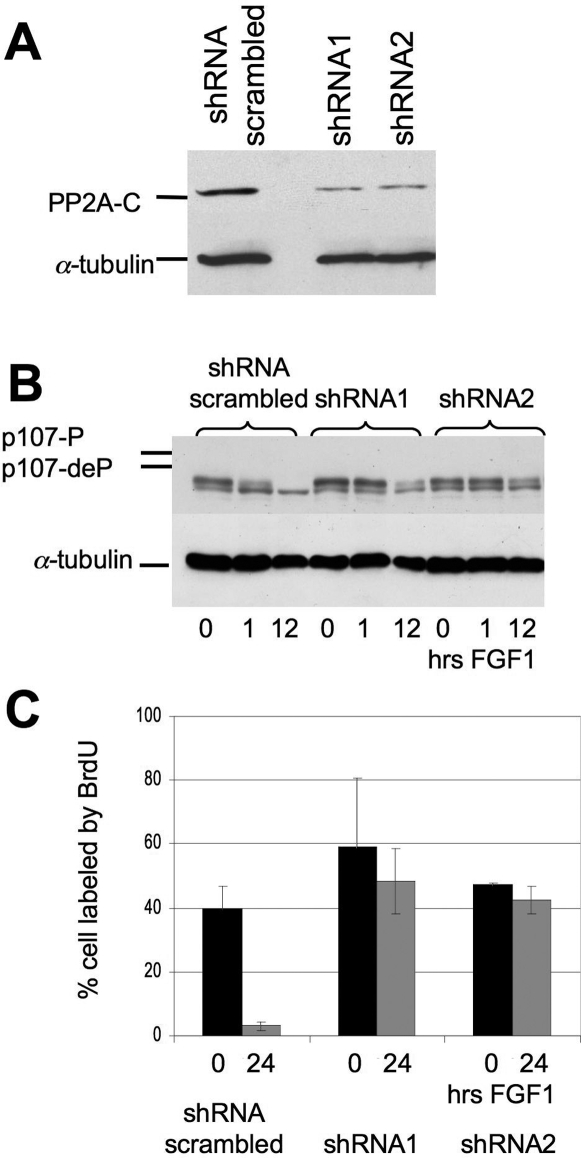
Knockdown of PP2A catalytic subunit inhibits FGF-induced p107 dephosphorylation. RCS cells were infected with retroviruses encoding either 2 different sequences specific for the α-isoform of PP2A-C subunit (shRNA1 and shRNA2) or scrambled shRNA and treated with FGF1. 20 µg of total protein were subjected to SDS-PAGE for inmmunodetection of (A) PP2A-C or (B) p107. (C) Percentage cells labeled by BrdU in the cell lines with knockdown of PP2A-Cα upon FGF treatment.

To confirm this conclusion, we sought to knockdown PP2A expression using RNA interference (Fig. 4). We infected cells with the pSilencer 5.1-U6 Retrovirus, containing shRNA sequences specific for the α isoform of the rat PP2A catalytic subunit. A comparable retrovirus expressing scrambled shRNA was used as control. Two sequences (shRNA 1 and 2, described in Materials and Methods) were found to reduce the expression of the PP2A-C protein by about 70% (Fig. 4A) and were used for further experiments. PP2A knockdown has been reported to trigger cell death in a variety of cell types [35], and we observed a similar effect in our experiments, precluding the isolation of cell clones with permanent PP2A knockdown. We therefore infected RCS cells with the shRNA retroviruses, selected shRNA expressing cells with puromycin for 3–4 days, and one day later determined PP2A-C levels and the cell response to FGF treatment.

p107 was dephosphorylated within the first hour following FGF treatment of cells expressing the scrambled shRNA. In contrast, knockdown of PP2A-C resulted in a substantial delay (up to 12 hrs) in p107 dephosphorylation (Fig. 4B). Furthermore, cells with reduced levels of PP2A-C retained about 80% of the original level of BrdU incorporating cells compared to about 10% in the control cell line (Fig. 4C), indicating that these cells are still progressing through the cycle even in the presence of FGF.

Although the results of PP2A-C knockdown were not as dramatic as those obtained with SV40ST, probably because the lethality of PP2A knockdown precluded the isolation of pure population of cells with substantially reduced levels of PP2A, the prevention of the FGF-induced p107 dephosphorylation and growth arrest we observed was significant and reproducible in independent experiments. We therefore conclude that PP2A function is required for the dephosphorylation of p107 and consequent growth arrest induced by FGF in RCS chondrocytes.

### FGF induces an association of PP2A with p107 in RCS chondrocytes

**Figure 5:**
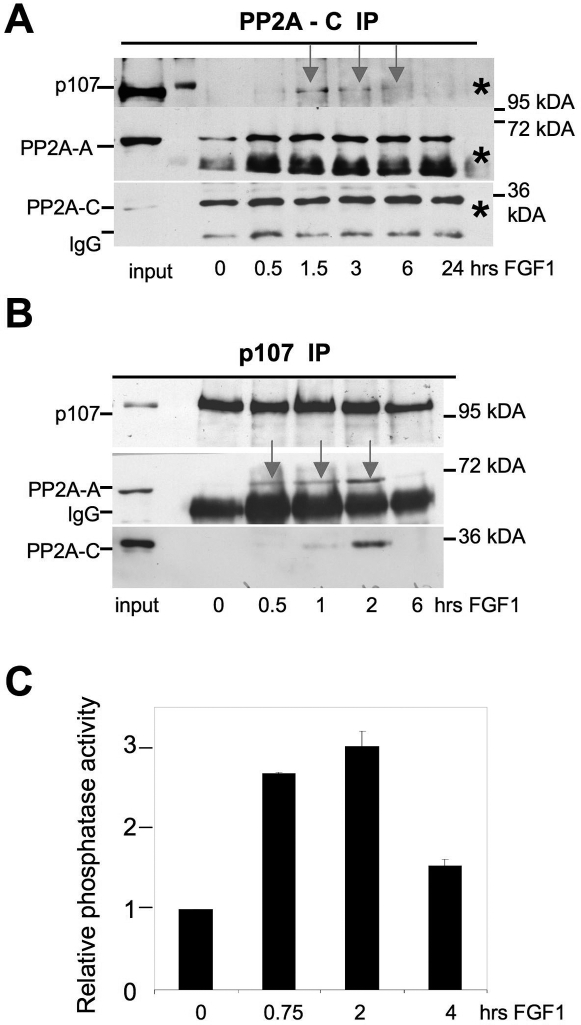
FGF induces an association between endogenous PP2A and p107. RCS cells were treated with FGF1 for the indicated periods of time. Lysates were prepared and normalized by the amount of total protein and subjected to immunoprecipitation with anti-PP2A-C (A) or anti-p107 agarose conjugated (B) antibody as described in Materials and Methods. 10% input and 50% IP samples were analyzed by WB with anti-PP2A-C, anti-PP2A-A or anti-p107 antibody. Control immunoprecipitation (*) was done using agarose A beads. (C) p107 immunoprecipitates from untreated and FGF treated RCS cells were used in *in vitro* Ser/Thr phosphatase assay. The background (protein A Sepharose without antibodies) was subtracted from initial counts and this value was normalized to one in the absence of FGF treatment.

To gain additional evidence for the hypothesis that FGF targets PP2A to p107 and causes p107 dephosphorylation, we immunoprecipitated either p107 or the PP2A-C subunit from FGF-treated RCS cells and determined whether an association between endogenous p107 and PP2A was induced by FGF treatment. Fig. 5A shows that while in untreated cells no association between p107 and PP2A could be detected, immunoprecipitation of PP2A-C from FGF-treated RCS brought down small but significant amounts of P107, most prominent at 1.5 and 3 hrs after FGF addition. As expected, PP2A-A subunits were also detected together with the C-subunits. Consistent with this result antibodies directed against p107 also immunoprecipitated PP2A-A and C subunits from FGF-treated cells but not from untreated cells (Fig. 5B). Interestingly, the association of PP2A with p107 induced by FGF is transient, and is almost undetectable after 6 hours of FGF treatment. This coincides with the increase in phosphatase activity that coimmunoprecipitates with p107 in FGF-treated cells, that also subsides at that time (Fig. 5C). This finding suggests that, although p107 is dephosphorylated rapidly and remains dephosphorylated for more than 24 hours after FGF treatment, the association of PP2A with p107 is transient and is no longer required to keep p107 in an hypophosphorylated state at later times, possibly because FGF signals are downregulated and/or the mechanisms leading to p107 phosphorylation are inactivated at such time.

Taken together these results and those discussed in the previous section support the hypothesis that in chondrocytes FGF signaling activates a known or yet to be discovered PP2A regulatory subunit, that would target PP2A to p107 . Indeed PP2A has been shown to promote myc dephosphorylation by a similar mechanism [36]. It is worth noting that the PR59/B subunit of PP2A has been reported to target PP2A to p107 [27], but our experiments have failed so far to detect an association between the PP2A-A and PR59 in both untreated and FGF-treated RCS cells. We were also unable to identify PR59 as part of the PP2A holoenzyme purified from RCS cells (data not shown). Recent work from Magenta et al. [30] has demonstrate that PP2A holoenzyme containing the PR70(PR48) subunit is responsible for pRb dephosphorylation and DNA synthesis inhibition induced by oxidative stress. However in our preliminary experiments the PR70 subunit was barely detectable in RCS cells and no association with p107 was detected either in FGF-treated or untreated cells (data not shown).

**Figure 6:**
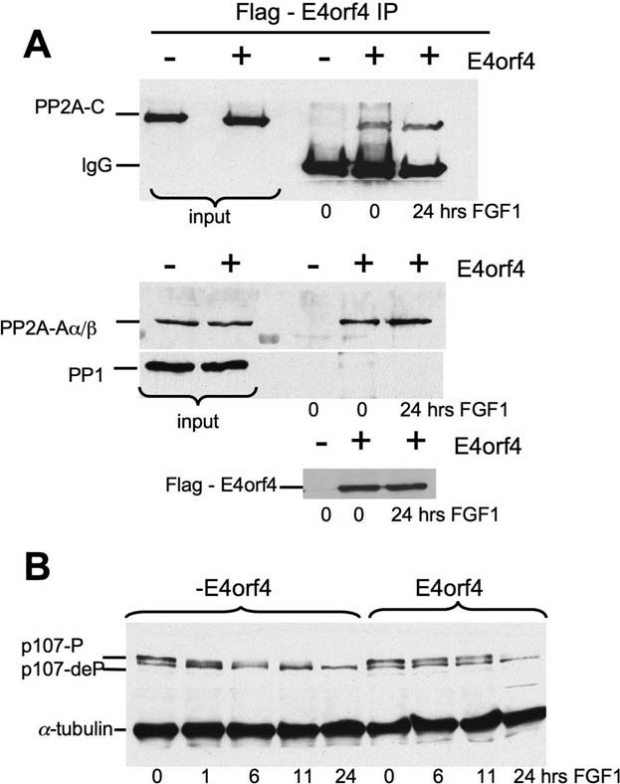
Expression of adenovirus E4orf4 protein strongly delays p107 dephosphorylation induced by FGF. FLAG-tag E4orf4 was stably introduced into RCS cells. Growing cultures were treated with FGF1 for indicated periods of time. (A) E4orf4 forms a stable complex with PP2A when overexpressed in RCS cells. RCS cells overexpressing FLAG-tag E4orf4 and the parental cell line were treated with FGF. E4orf4 was immunoprecipitated with ANTI-FLAG® M2-Agarose and immunoprecipitated complexes were analyzed using PP2A-A, PP2A-C or FLAG antibodies as indicated. PP1 antibody was used as a negative control. As a reference 5% of the immunoprecipitated whole cell extract (“input”) was loaded. (B) 20 µg of total protein were subjected to immunodetection by p107 antibody.

In an attempt to narrow down the search for the putative regulatory B subunit activated by FGF, we have expressed the adenovirus E4orf4 protein, which is thought to interact with a subset of intact PP2A holoenzymes that contain either B55α or B56 family members [37], [38] in RCS cells (Fig. 6). Coimmunoprecipitation experiments showed that the E4orf4 protein formed stable complexes with the PP2A A and C subunits (Fig. 6A). In FGF-treated RCS cells overexpressing E4orf4, p107 dephosphorylation was delayed up to 11 hrs, while in parental RCS cells p107 was completely dephosphorylated in 1 hr (Fig. 6B). However, by 24 hrs the dephosphorylation of p107 in the presence of E4orf4 was complete and cell proliferation was arrested (data not shown). These experiments therefore suggest that B55α or B56 containing PP2A complexes may be specifically involved in p107 dephosphorylation, but the mechanism of action of E4orf4 is not completely understood, and some general impairment of PP2A function cannot be excluded. Thus while these results are consistent with the hypothesis that PP2A function is required for p107 dephosphorylation, the identification of the specific regulatory subunit involved will require further studies.

## Discussion

The results presented in this report are consistent with the notion that the dephosphorylation of p107 induced by FGF in chondrocytes initiates a cascade of events that leads to inhibition of cell proliferation. Preventing p107 dephosphorylation in FGF-treated cells, whether by overexpression of a p107 kinase or by inactivation of the PP2A phosphatase, also prevents FGF-induced cell cycle arrest, dephosphorylation of p130, and downregulation of cyclin E/cdk2 and cyclin A/cdk2 activity. The finding that preventing p107 dephosphorylation by overexpression of the cdk4/cyclinD complex also results in higher cdk2/cyclinE activity is consistent with the hypothesis that the activation of p107 by FGF signaling induces its association with E2F4 to produce transcriptionally repressive complexes that inhibit the expression of genes required for cell cycle progression [39]. Since both cdk2 and cyclinE have been reported to be E2F targets [40], [41], the downregulation of cyclinE/cdk2 expression and/or activity in FGF-treated chondrocytes could result from the activation of p107. The inhibition of p107 dephosphorylation by D1/cdk4 overexpression would thus result in maintaining high levels of cyclinE/cdk2 activity and consequently p130 phosphorylation. Additionally, the overexpressed cyclin D1 and cdk4 could sequester p21 and p27, therefore reducing the repression of the cyclin E/cdk2 activity by these kinase inhibitors. Indeed it has been recently reported [42] that mice which are mutant for both p107 and p27 function display a phenotype of exaggerated chondrocyte proliferation very similar to that of p107/p130 knockouts, supporting the notion that the increase in p130 phosphorylation we observe by preventing p107 dephosphorylation would result from enhanced cdk2/cyclin E activity, and that p130 reinforces the cell cycle exit decision made by p107.

In conclusion, p107 dephosphorylation appears to be an early and necessary event in the induction of cell cycle arrest by FGF. These results identify p107 dephosphorylation as a decisive event in FGF-induced growth arrest, and indicate that FGF signaling does not directly target downstream effectors of Rb protein function, such as E2F1 or cyclin E. Interestingly, these phenomena are cell type-specific. In fibroblasts, where FGF signaling is a potent mitogen, FGF does not induce p107 dephosphorylation and actually increases its phosphorylation when applied to quiescent cells.

It is clear however that FGF signaling utilizes multiple mechanisms to inhibit chondrocyte proliferation, most of which are probably necessary but not sufficient to induce growth arrest [43]. FGF signaling induces the expression of cdk inhibitors, particularly p21 but also p27 and p57, and the induction of these cell cycle inhibitors is likely to contribute to the FGF-induced growth arrest, and to Rb protein dephosphorylation. However these events occur late after FGF treatment, after p107 dephosphorylation has already taken place, require new RNA and protein synthesis, and thus are likely to play a role in the late stages of the FGF response, that we had defined as “maintenance of growth arrest”[3]. STAT1 and AKT activities are also important for FGF-induced growth inhibition [44], [45]. However, also in this case our results indicate that the roles played by STAT1 and AKT in this process are directed toward the late stages of the FGF-response. Clearly, elucidating the complex and probably multiple mechanisms by which FGF inhibits chondrocyte proliferation and the cross-talk between these seemingly independent pathways will require further studies.

Another novel and interesting finding was that inhibiting the function of PP2A, whether by expression of the SV40 ST or by knockdown of the PP2A catalytic subunit by shRNA, prevented FGF-induced dephosphorylation of p107 and cell cycle arrest. Together with the results showing that FGF induces an association between PP2A and p107 in RCS cells, these findings suggest that FGF signaling activates PP2A to target p107 for dephosphorylation. It has been shown that PP2A can dephosphorylate p107 in vitro [27], but the regulation of PP2A activity is very complex and still not fully understood [31]. It is interesting to note that the association of p107 with PP2-A that is induced by FGF is transient, and limited to the first 5–6 hours of FGF treatment. This suggests that, once dephosphorylated, p107 is no longer a target for PP2A and that the perturbation of cell cycle progression induced by p107 dephosphorylation is no longer reversible. PP2A has been previously implicated in Rb protein dephosphorylation, particularly under conditions characteristic of stress response (UV, H_2_O_2_, etc.), and thus it is possible that PP2A may not be the phosphatase that normally dephosphorylates p107 in the cell cycle, but it could be recruited for this task only under special conditions. At this point the Ser/Thr residues that are initially dephosphorylated may not be the same that are dephosphorylated during the normal cell cycle [46] and the kinetics of dephosphorylation could also be different.

It could be argued that PP2A has been shown to regulate many signal transduction pathways and it could play a role in other processes that are important for p107 dephosphorylation. Thus the effect of inhibiting PP2A function on p107 dephosphorylation could be indirect. There are many growth-related processes in which PP2A appears to play a role, including the regulation of the G2-M transition, control of translation, c-myc stability, ERK1/2 activation, AKT phosphorylation and Wnt signaling [31]. However the cell cycle block induced by FGF in chondrocytes affects mainly the G1/S transition, overall translation and c-myc expression are not affected until very late after FGF treatment, and the inhibition of PP2A activity would be expected to increase ERK1/2 activation, while ERK1/2 inhibition reduces p107 dephosphorylation and the FGF-induced growth arrest [21]. Thus the only known effect of PP2A that could conceivably impact on the growth-inhibitory response of chondrycytes to FGF is its down-regulation of AKT phosphorylation. AKT activity is reduced in FGF-treated RCS cells and expression of a constitutively activated AKT partially relieves the FGF growth inhibitory response [45]. However the growth-promoting effect of activated AKT in RCS chondrocytes is minimal when compared to the effect of PP2A inhibition, and does not influence p107 dephosphorylation. Furthermore, we did not detect any increase in AKT phosphorylation in RCS cells expressing PP2A-specific shRNA when compared to controls, with or without FGF treatment. Thus the simplest interpretation of our results is that FGF treatment of chondrocytes results in the recruitment of PP2A to perform p107 dephosphorylation.

**Figure 7:**
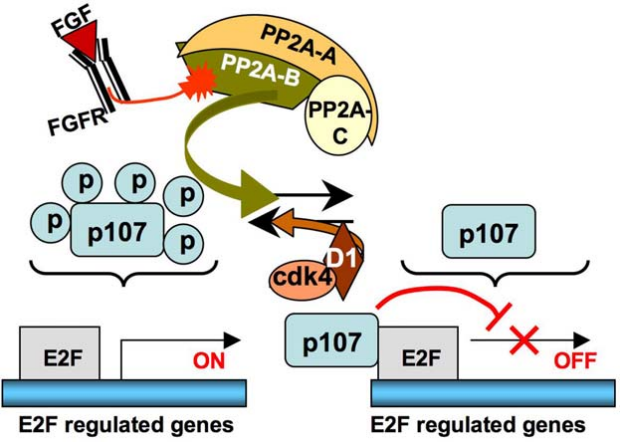
A model for FGF regulation of p107 phosphorylation in RCS cells. FGF signaling activates a regulatory B subunit that targets the PP2A holoenzyme to p107.

In conclusion, our results identify a novel role of PP2A on regulation of chondrocyte proliferation, that intervenes early and upstream of p107 dephosphorylation to mediate FGF-induced growth arrest. We are investigating the hypothesis that FGF “activates” a specific regulatory subunit of PP2A that would target this phosphatase to p107 (Fig. 7). The identification of this regulatory subunit and its mechanism of activation by FGF may provide important clues for the understanding of PP2A regulation as well as of the cell type-specific mechanisms that distinguish the FGF response of chondrocytes from that of other cell types.

## Materials and Methods

### Cell culture, proliferation assay and FACS analysis

Rat chondrosarcoma (RCS) cells [47] were maintained in DMEM supplemented with 10% fetal calf serum at 37°C and 9% CO_2_. Cells were treated with FGF1 (5 ng/ml) (a kind gift from M. Mohamadi, NYU) and heparin (5 µg/ml) in the absence of antibiotics. For proliferation assay RCS cells were incubated for 2 hrs in the presence of 2 µg/ml of BrdU. Incorporation of BrdU and FACScan analysis of propidium iodide stained cells were performed as described previously (Laplantine, et al., 2002).

### Plasmids

Rat cyclin D1 was introduced into pLXSN vector between *EcoRI* and *BamH*1 sites. Cdk4/pBABEpuro construct was made by inserting the PCR product into pBABEpuro vector between *BamH*1 and *EcoRI* sites. The PCR product coding human cdk4 was obtained from pCMVcdk4 plasmid (a kind gift from M. Pagano, NYU) using primers 5′-ATATGGATCCACCATGGATTACAAGGACGACGATGACAAGATGGCTACCTCTCGATATGAGCCAGTGGC-3′ and 5′-TATAGAATTCTCACTCCGGATTACCTTCATCCTTAT-3′. (FLAG-tag is underlined). Plasmids containing mutant forms of cdk4 were generated using “Quik-Change Site-Directed Mutagenesis Kit” (Stratagene) on the base of cdk4/pBABEpuro plasmid as specified by the manufacturer. shRNA constructs were made according to manufacture procedure (Ambion), using p*Silencer*™ 5.1-U6 Retro vector. shRNA1 and shRNA2 target sequences which are complementary to nucleotides 465–483 and 299–317 of rat PP2A catalytic subunit α-isoform (NCBI accession #NM_017039). E4orf4-FLAG construct was obtained from FLAG.E4orf4/pcDNA3 vector (a kind gift from Josee N. Lavoie, University Laval, Quebec) by inserting the PCR product into pBABE-puro vector between the BamHI and EcoRI sites.

### Retroviral and adenoviral infection

Retroviral infection was performed as described previously [21]. For adenoviral infection, chondrocytes were trypsinized, resuspended in TRIS buffer at final concentration of 2×10^6^ cells/ml and exposed to 10 pfu/cell of Ad-GFP, Ad-ST SV40 or Ad- ST SV40 mutant Cys97Ser in suspension for 1 hr at room temperature. After 20 hrs cells were treated with FGF and heparin as indicated.

### Immunoprecipitation, Western blot analysis and *in vitro* kinase and phosphatase assays

Protein lysates were prepared using modified RIPA buffer (50 mM Tris HCl pH 7.4, 150 mM NaCl, 10 mM KCl, 1% NP-40, 1 mM EDTA) in the presence of 1 mM Na_3_VO_4_, 10 mM NaF, 1 mM PMSF and 5 µg/ml aprotinin. For immunoprecipitation 0.5 mg of total protein was pre-cleared by incubating for 30 min at 4°C with Protein G-Sepharose® 4B Conjugate (ZYMED), and then incubated with 2 µg of antibody overnight at 4°C. Protein G-Sepharose was added for 1 h at 4°C and the immune complexes were washed 3 times with 1 ml of modified RIPA buffer. Immunoprecipitates with antibodies against p107 and PP2A-C catalytic subunit were prepared from 1 mg of total protein and 5 µg of antibody. NaF was omitted from RIPA buffer for these experiments. Protein A-Sepharose was added when anti-PP2A-C antibodies were used. Immunoprecipitates were resolved on SDS-PAGE and analyzed by immunoblotting. The following antibodies were used: anti-α-actin (AC-40), anti-α-tubulin (clone B-5-1-2) and anti-FLAG M2 (Sigma), anti-p130 (BD Transduction Laboratories), anti-cyclinD1, anti-PP2A-A subunit and anti-phospho p42/p44 MAP kinase (Cell Signaling), anti-cdk4 (Chemicon International), anti-ST SV40 (Calbiochem), anti-p107 (C-18), anti-p21 (C-19), anti-cyclin E (M-20), anti-cyclin D1 (72-13G) anti-cyclin A (H432), anti-cdk2 (M2) (Santa Cruz Biotechnology), anti-PP2A-C subunit (Millipore). The determination of cyclin E/cdk2 and cyclin A/cdk 2 activities on histone H1 was done using anti-cyclin E and anti-cyclin A antibodies as described previously [3] . Immunoprecipitates for protein Ser/Thr phosphatase assay (New England BioLabs) were prepared as described above for the co-immunoprecipitation experiments. After the final wash, immunoprecipitates were washed once more with the phosphatase assay buffer (50 mM Tris HC1 pH 7.4, 0.1 mM Na_2_EDTA, 5 mM DTT, 0.0% Brij 35) and the pellet was resuspended in the same buffer (40 µl total volume). 10 µl of labeled substrate (myelin basic protein) was added to initiate reaction. Reaction mixtures were incubated for 10 min at 30°C. The reactions were terminated by adding 200 µl of cold 20% TCA and incubated 15 min on ice. After 5 min centrifugation at 12,000×g 200 µl of the TCA supernatant were counted in 5 ml of scintillation fluid. The background (protein A Sepharose without antibodies) was subtracted from initial counts and the values obtained were normalized to the results obtained in the absence of FGF treatment.

## Supporting Information

Figure S1 FGF-induced p107 dephosphorylation and ERK1/2 activation in primary chondrocytes. Chondrocytes were isolated from femoral and tibial growth plates of 10-day old mice, plated in 6-well plates and after 48 hours were treated with FGF1 for 2 hours (10 ng/ml). The cells were collected directly in 300 µl 2XSDS buffer and analyzed by SDS-PAGE followed by immunoblotting against anti-p107 and anti-phospho-ERK1/2 antibodies. Hyper-and hyposphosphorylated forms of p107 are indicated. Equal amount of protein loading was confirmed by α-tubulin immunodetection.
(5.70 MB TIF)
