# Supplementary figures and images for: PP2A-Mediated Dephosphorylation of p107 Plays a Critical Role in Chondrocyte Cell Cycle Arrest by FGF
Source: PLoS One. 2008 Oct 17;3(10):e3447. doi: 10.1371/journal.pone.0003447 (PMC2562983; doi:10.1371/journal.pone.0003447)

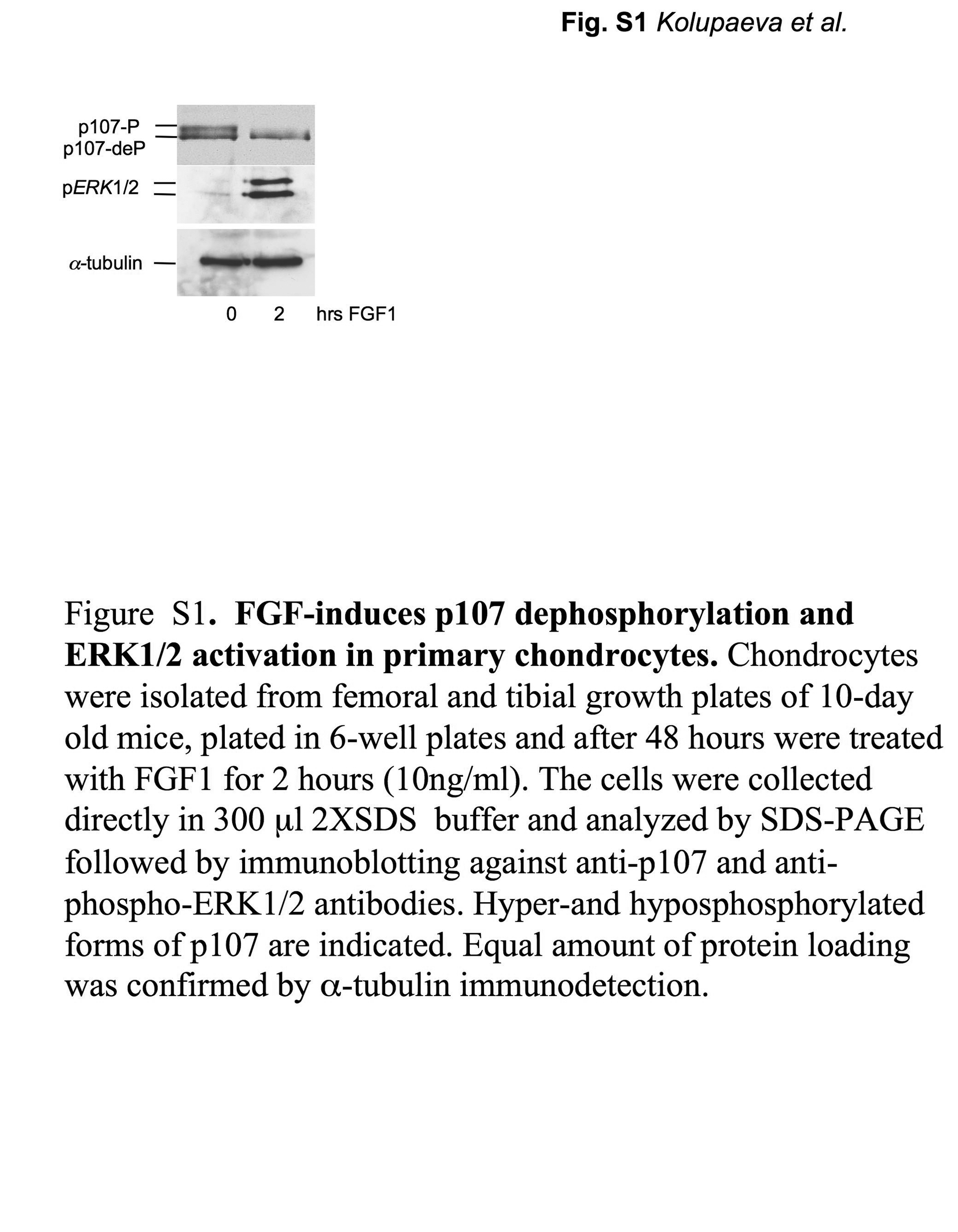

Supplement: Figure S1 — FGF-induced p107 dephosphorylation and ERK1/2 activation in primary chondrocytes. Chondrocytes were isolated from femoral and tibial growth plates of 10-day old mice, plated in 6-well plates and after 48 hours were treated with FGF1 for 2 hours (10 ng/ml). The cells were collected directly in 300 µl 2XSDS buffer and analyzed by SDS-PAGE followed by immunoblotting against anti-p107 and anti-phospho-ERK1/2 antibodies. Hyper-and hyposphosphorylated forms of p107 are indicated. Equal amount of protein loading was confirmed by α-tubulin immunodetection. (5.70 MB TIF) [file pone.0003447.s001.tif]
